# Targeting MITF as a tyrosine-kinase-inhibitor-independent strategy for treating GIST

**DOI:** 10.1016/j.omton.2025.201039

**Published:** 2025-09-08

**Authors:** Sai Fung Yeung, Qiqian Huang, Kaixi Liang, Stephen Kwok Wing Tsui

**Affiliations:** 1 School of Biomedical Sciences, The Chinese University of Hong Kong, Hong Kong SAR, Hong Kong

Gastrointestinal stromal tumors (GISTs), the most common GI mesenchymal neoplasms, are molecularly defined by mutually exclusive driver mutations, primarily in *KIT* (60%–70%), *PDGFRA* (10%–15%), or *SDH* complex genes (15%). While no targeted therapies are currently approved for SDH-deficient GISTs, approximately 80% of cases are targetable by *KIT/PDGFRA* tyrosine kinase inhibitors (TKIs). Consequently, genotype-directed therapy is established as the cornerstone of management. Since the early 2000s, imatinib revolutionized the treatment of advanced GISTs, extending median overall survival from ∼1 year to 4–5 years with response rates >50%. To date, it remains the first-line therapy for most *KIT/PDGFRA*-mutant GISTs.^1^ However, resistance persists: primary resistance occurs in *PDGFRA* D842V-mutant and *SDH*-deficient tumors (response <15%), while secondary resistance—driven by acquired kinase mutations, tumor heterogeneity, or adaptive mechanisms—typically causes relapse within 2–3 years. Later-generation TKIs (sunitinib, regorafenib, ripretinib, avapritinib) address specific mutations but often yield only transient benefit.^2^ These limitations underscore the need for novel strategies beyond sequential kinase inhibition.

To address this gap, emerging approaches now include TKI-centric optimization through novel agents (e.g., pan-KIT mutant inhibitor AZD3229) and rational combination TKI regimens that expand mutants coverage. Concurrently, non-kinase targeting strategies are gaining momentum: tumor-selective antibody-drug conjugates (e.g., anti-GPR20 monoclonal antibodies delivering cytotoxic payloads); radioactive labeled tumor-specific ligands (e.g., ^177^Lu-NeoB radiolabeled peptides for precision delivery of radiotherapy); temozolomide for *SDH*-deficient GISTs; and TKI-immunotherapy hybrids to enhance checkpoint inhibition.^3^

In this issue, Guerrero et al.^4^ report direct targeting of microphthalmia-associated transcription factor (*MITF*) with the small molecule inhibitor ML329, demonstrating suppression of survival in both imatinib-sensitive and imatinib-resistant GIST models. In this commentary, we explore *MITF* as a lineage-survival vulnerability in GIST and consider a broader implication of targeting transcription factor (TF) across oncology.

*MITF* is a basic-helix-loop-helix leucine zipper TF that is essential to the melanocyte lineage. It controls key genes involved in pigmentation (*TYR*, *TYRP1*, and *DCT*), cell-cycle progression (*CDK2* and *CCND1*), apoptotic (*BCL2* and *ML-IAP*), metabolism, and autophagy (*PGC1A*, *LC3B*, and *SQSTM1*). In melanoma, *MITF* acts as a crucial lineage-survival oncogene, where high expression is associated with stem-like features, drug resistance, metastatic potential, and an immunologically “cold” tumor microenvironment.^5^ MITF oncogenic role also extends to clear cell renal cell carcinoma (ccRCC), renal angiomyolipoma, non-small cell lung cancer (NSCLC), and gastrointestinal stromal tumors (GISTs).^5^

Previous work by Proaño-Pérez et al.^6^ demonstrated GISTs are dependent on *MITF* for survival. *MITF-A* was identified as the dominant isoform in GIST, driving survival and proliferation through activation of *KIT*, *BCL2*, and *CDK2*. Silencing *MITF-A* induces cell cycle arrest and impaired tumor growth both *in vitro* and *in vivo*, in part by disrupting a regulatory circuit involving *ETV1*, a lineage-survival factor directly regulated by KIT signalling.^6^ A high-throughput screening identified ML329 is a selective, direct inhibitor of *MITF*,^7^ which has been documented to inhibit MITF-dependent melanoma cells.^8^

Based on these findings, Guerrero et al.^4^ showed that ML329 profoundly impacts both imatinib-sensitive (GIST-T1) and imatinib-resistant (GIST430/654) GIST cell survival. ML329 suppresses proliferation, downregulates *MITF* target genes, and induces S to G2/M phase cell-cycle arrest. ML329 is well tolerated *in vivo*, significantly reduces tumor growth, and improves overall survival. Although it shows only modest synergy with imatinib, ML329 demonstrates weak but notable synergy with ripretinib in resistant models, suggesting potential for rational combination strategies. Mechanistically, ML329-induced cell death likely involves ferroptosis, as indicated by the rescue of cell viability with deferoxamine mesylate. This suggests that the induction of ferroptosis may be a mechanism of ML329 action.^4^ Given that TKI resistance often arises from secondary *KIT* or *PDGFRA* mutations and broader tumor heterogeneity, combining ML329 with other treatment modalities could offer enhanced efficacy in advanced GIST.

TF dysregulation is a hallmark of many human cancers. The findings above highlight MITF as a clinically actionable vulnerability in GIST, illustrating the broader therapeutic potential of targeting TFs. With ∼1,600 TFs encoded in the human genome, many (such as *MYC*) act as master regulators of oncogenic signaling and treatment resistance. Unlike kinases, TFs were long deemed “undruggable” due to barriers such as intrinsically disordered structures, lack of defined binding pockets, structural flexibility, and nuclear localization. However, advances in structural biology, chemical biology, drug discovery, and AI-driven design are now overcoming these barriers.

Historically, nuclear hormone receptors estrogen receptor (*ER*) and androgen receptor (*AR*) are ligand-activated TFs and were among the first successful oncology targets. Competitive antagonists (tamoxifen, flutamide, and bicalutamide) and hormone depletion (surgical or medical castration) provided a founding model for ligand-modulated TF inhibition. While some TFs require only a DNA-binding domain and an effector domain to recruit the transcriptional machinery, others exhibit far more complex regulatory structures. For instance, *MYC* contains a C-terminal helix-loop-helix leucine zipper domain that necessitates dimerization with *MAX* to bind E-box sequences.^9^^,^^10^ As such, diverse approaches were tailored to the structural and functional properties depending on individual TFs (Figure 1).

**Figure 1:**
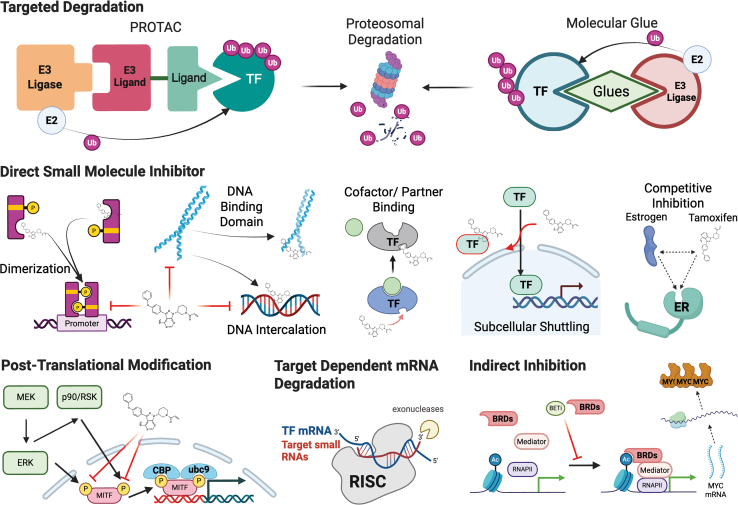
Schematic overview of strategies for targeting transcription factors

Direct inhibitors of structured domains such as ligand-binding pockets, DNA-binding motifs, or protein–protein interaction interfaces—exert their effects by binding to these regions and disrupting critical molecular interactions. For example, iCRT3 disrupts the β-catenin–TCF complex, thereby downregulating target genes such as *CCND1* and *MYC*. However, many TFs lack well-defined binding pockets, and direct inhibitors often suffer from limited specificity. An alternative strategy involves indirect inhibition of upstream regulators, such as kinases or epigenetic readers. Notably, BET bromodomain inhibitors like JQ1 and I-BET interfere with *BRD4* occupancy at *MYC* super-enhancers, leading to transcriptional repression of *MYC*. Exploiting post-translational modifications represents another TF-targeting strategy. For instance, inhibition of the *MAPK* pathway with MEK inhibitors such as trametinib can reduce *MITF* phosphorylation (S73/S409) by *ERK/RSK* and suppress its transcriptional activity. A more recent development to TF targeting involves the use of targeted degraders. These molecules link a TF to a degrader like E3 ligase complex, promoting its ubiquitination and proteasomal or lysosomal degradation. Technologies such as PROTACs, molecular glues, TRAFTACs, and pepTACs exemplify this strategy. For example, ARCC-4 recruits *VHL* to degrade *AR* in prostate cancer cells.^9^^,^^10^

Taken together, these findings establish MITF as a clinically actionable vulnerability in GIST that circumvents TKI resistance. Targeting transcription factors represent a promising strategy to broaden therapeutic options and improve outcomes. Clinical translation will require thorough safety assessment of ML329 and validation in patient-derived xenografts and organoids. Further research on rational combinations of MITF inhibitors with kinase inhibitors, immunotherapies, or protein degraders could yield durable, precision therapies for GIST.

## Acknowledgments

The authors would like to acknowledge the funding from the Research Impact Fund (#R4015-19) and the Theme-based Research Scheme (T12-716/22-R) from the Research Grants Council of the Hong Kong Special Administrative Region. Figure was created in https://BioRender.com.

## Author contributions

Conceptualization, S.F.Y.; writing, S.F.Y. and Q.H.; review, comment, and editing: K.L.; senior supervision and funding, K.W.T.

## Declaration of interests

The authors declare no conflicts of interest.
